# The Mechanical Properties, Corrosion Resistance, and Biocompatibility of a Novel Ternary Ti-xNb-5Ta Alloy for Biomedical Applications

**DOI:** 10.3390/ma18030602

**Published:** 2025-01-28

**Authors:** Haochen Wu, Tao Jiang, Linghui Kong, Xiaohong Chen, Ping Liu

**Affiliations:** School of Materials and Chemistry, University of Shanghai for Science and Technology, Shanghai 200093, China

**Keywords:** ternary alloy, near-β-type titanium alloy, biocompatible materials, corrosion resistance, early osteogenic differentiation

## Abstract

In recent years, advancements in dental implants have posed new challenges for the use of traditional orthopedic materials. This study prepared novel Ti-xNb-5Ta alloys via arc-melting and comprehensively evaluated the effects of Nb content on microstructure, mechanical properties, elastic modulus, electrochemical behavior, and in vitro performance. Interestingly, alloys with different Nb contents exhibited distinct properties. The results indicated that the 10 and 13 wt.% Nb alloys surpassed the TA4G surgical implant standard in strength while offering a lower elastic modulus and greater elongation. Electrochemical experiments showed that the corrosion resistance of the alloys improved with increasing Nb content. Furthermore, CCK-8 assay results, ALP semi-quantitative analysis, and RT-PCR demonstrated that Ti-xNb-5Ta alloys enhanced the early osteogenic differentiation of human bone marrow stromal cells (hBMSCs). This work not only reveals the potential of Ti-xNb-5Ta alloys as biomedical materials but also offers insights for developing novel biomaterials.

## 1. Introduction

Over the past decade, advancements in medical technology have significantly increased the average human life expectancy, leading to a pronounced aging population trend in countries such as Japan, Italy, and Finland [1]. Simultaneously, the frequent outbreaks of global diseases such as COVID-19 and monkeypox have heightened public awareness of health [2]. These factors collectively drive the increasing demand for biomedical materials, positioning them as a burgeoning research field. Biomedical materials offer essential support under extreme conditions and provide critical alternatives at vital moments, with the potential to extend the human lifespan further [3]. They hold immense promise in advancing early and precise disease diagnosis, enabling the functional replacement of teeth and bones, fostering innovations in surgical implant technologies, and promoting the regeneration and repair of damaged organs [4].

Metallic materials are a type of biomaterial that meets most clinical requirements in orthopedic implants. Currently, CP-Ti is the predominant metallic material used in implants [5]. However, its strength of 265–353 MPa falls short in extreme conditions, leading to complications or adverse reactions after implantation. For example, severe atrophic alveolar ridge dental implant surgery requires narrow-diameter implants, which have a 7.6% failure rate in 3 years [6], but the expected failure rate is less than 3% [7]. Unexpected failure rates are due to two main reasons: one is due to the implant’s mechanical properties and the other is peri-implantitis at the bone–implant interface [8]. Therefore, a range of materials have been developed. Lin et al., using a cathodic hydrogen charging method, prepared a 33.97 GPa Young’s modulus hydrogenated Ti-6Al-4V alloy [9]. Chen et al. prepared a tantalum gyroid porous scaffold with an elasticity modulus similar to human cancellous bone, ranging from 215 to 1103.67 MPa via additive manufacturing [10]. Saha et al. explored Co-Cr alloy corrosion performance in artificial saliva, and the results demonstrated that the alloy had long-term viability [11]. All of the above alloys offer superior properties compared to CP-Ti [12]. However, these materials still face several challenges. In Ti-6Al-4V, vanadium and aluminum can be toxic during electrochemical corrosion. Vanadium potentially leads to bone resorption, and aluminum may cause some neurological conditions such as Alzheimer’s disease [13,14]. Co-Cr alloys can release toxic Co and Cr ions during corrosion [11]. Although tantalum is a highly biocompatible metal, Jin et al. pointed out that tantalum has the potential ability to reduce the occurrence of peri-implantitis [15]. However, tantalum is prohibitively expensive and challenging to process due to its high melting point. Furthermore, it is commonly used as an auxiliary element in alloys or for fabricating porous scaffolds [16].

Moreover, the stiffness currently used in dental implant Ti alloys is 3–4 times higher than that of human bone [17]. This mismatch leads to a stress shielding effect, where the implant and jawbone deform inconsistently under stress, potentially resulting in osteoporosis or bone resorption over time. In extreme conditions, this unbalanced relationship will cause implant or bone-breaking [18,19,20,21]. In addition, Ti alloys are passivating metallic materials, and the corrosion resistance and stability of the passive film on the implant surface are critical for osseointegration and bone cell adhesion, which directly affect the material’s biocompatibility [22]. The addition of alloying elements can help to give the passivated film better biocompatibility or antibacterial ability.

Nowadays, integrating non-toxic elements into titanium alloys has become a hotspot of research on biomaterials, such as Nb, Ta, Zr, Cu, and Mg [23]. These elements contribute to the biocompatibility and excellent corrosion resistance of the alloy. Generally, the Ti-Nb alloy has a low elastic modulus, and we can add different elements such as Ta, Zr, and Mo to target the weak point of the Ti-Nb system and prepare new alloys [24].

In conclusion, there is an urgent need to develop a novel, non-toxic, titanium alloy that can meet the clinical demands of dental implants. This study investigated a series of ternary Ti-xNb-5Ta (x = 5, 7, 10, 13 wt.%) titanium alloys, which, to our knowledge, have not been previously reported. Our research aims to unveil a potential range of properties of these alloys. The findings will lead to the development of advanced titanium alloys for future biomedical implants.

## 2. Materials and Methods

### 2.1. Alloy Design

Among the metal materials commonly used in biomedicine, the molybdenum equivalent (Mo_eq_), defined in Equation (1), is a widely accepted empirical parameter for determining the amount of β stabilizer. Generally, a higher Mo_eq_ leads to a more stable β phase in multi-component titanium alloys. Metastable β-type or near-β-type titanium alloys typically have Mo_eq_ values between 2.8 and 23, while high-performance medical titanium alloys have Mo_eq_ values ranging from 2.8 to 17.7 [25].

(1) $$\begin{array}{c} {\mathrm{Mo}}_{\mathrm{eq}}=1.0Mo+0.67V+0.44W+0.28Nb+0.22Ta+2.9Fe\\ +1.6Cr-1.0Al-0.33Sn-0.17Zr\left(wt.\%\right) \end{array}$$

The d-electron theory was also used to effectively design titanium alloys [26,27]. Generally, higher bond orders (B_o_) enhance solid solution strengthening, while a lower metal d-orbital energy level (M_d_) contributes to phase stability. For biomedical titanium alloys, the B_o_ value typically ranges from 2.75 to 2.85 and the M_d_ value ranges from 2.35 to 2.45 eV [28]. This study focused on novel near-β-type Ti-xNb-5Ta alloys with Mo_eq_ values of 2.50, 3.05, 3.88, and 4.72. The average B_o_ values were 2.803, 2.807, 2.813, and 2.818 and the average Md values were 2.448, 2.447, 2.446, and 2.446 eV, respectively. Based on the phase stability diagram of B_o_ and M_d_ summarized by Sidhu [29], the alloys designed in this study corresponded to the primary α″ phase.

### 2.2. Ingots Preparation

Ti-xNb-5Ta alloy ingots were prepared via arc-melting (model Arc Melter AM 200, Edmund Bühler GmbH, Bodelshausen, Germany) using a mixture of high-purity Ti (99.99 wt.%, Aoshuo, Hebei, China), Nb (99.99 wt.%, Aoshuo, Hebei, China), and Ta (99.99 wt.%, Aoshuo, Hebei, China) in an argon (Honghui, Jiangsu, China) atmosphere. Each ingot was remelted five times in the furnace during a single melting process to ensure compositional uniformity. After melting, the actual composition of the ingots was measured according to the GB/T 4698 standard [30]. The specific composition is detailed in Table 1.

### 2.3. Specimen Characterization

Before microstructural characterization, the samples were mirror-polished according to the guidelines by Gammon [31]. Final polishing was performed using 0.05 μm alumina metallurgical polishing powder on a polishing cloth. Where necessary, Kroll’s reagent was applied as an etchant to reveal the microstructure for optical microscopy (OM, model DMi8A, Leica, Wetzlar, Germany). The microstructural constituents of the material were examined using X-ray diffraction (XRD, model D8 Advance, Bruker, Billericca, MA, USA). The tensile strength and elongation of the alloys were determined using an electronic universal material testing machine (model UTM5504, Sansi, Shenzhen, China). The tensile properties and elastic modulus were assessed via tensile testing, while the elastic modulus was calculated using the tensile method. Vickers microhardness was determined using a Vickers hardness tester (model HVS-1000Z, Truer, Shanghai, China) at a load of 1000 gf for 10 s.

### 2.4. Electrochemical Experiment

Corrosion performance was evaluated using open circuit potential (OCP), polarization curve, and electrochemical impedance spectroscopy (EIS). A conventional three-electrode electrochemical cell system (model CHI660E, Chenhua, Shanghai, China) was used, with a saturated calomel electrode (SCE) as the reference electrode, platinum as the counter electrode, and Ti-xNb-5Ta as the working electrode with an exposed area of 0.78 cm². Each sample was immersed in 200 mL of simulated body fluid (SBF, chemical composition listed in Table 2) with a stabilized pH of 7.45 in equilibrium with air. The tests were conducted at room temperature under static conditions, with three measurements performed per sample to ensure statistical validation.

### 2.5. Surface Bioactivity Assessment

In order to evaluate surface biomineralization potential, the samples were immersed in an SBF solution to examine the formation of calcium/phosphorus compounds on their surfaces. The samples were positioned vertically in centrifuge tubes filled with SBF and incubated at 37 °C. The SBF solution was refreshed every three days. After 28 days, the samples were removed, gently rinsed with deionized water, dried at 40 °C for one hour, and coated with gold. Surface morphology and elemental composition were analyzed using scanning electron microscopy (SEM, model Quanta FEG-450, FEI, Hillsboro, OR, USA).

### 2.6. Cytocompatibility and Osteogenic Differentiation Assay

The cytocompatibility and osteogenic potential of Ti-xNb-5Ta alloys were evaluated using hBMSCs (Saibaikang, Shanghai, China) on samples with a diameter of 10 mm. Cytotoxicity was assessed through a CCK-8 assay and cell morphology on sample surfaces was observed using confocal laser scanning microscopy (CLSM). Alkaline phosphatase (ALP), a recognized osteoblast marker, was measured in hBMSCs cultured on Ti-xNb-5Ta alloys, with semi-quantitative determinations conducted on days 5 and 10. After 7 days of induction, total RNA was isolated and reverse-transcribed into cDNA for RT-PCR amplification, with primer sequences provided in Table 3.

## 3. Results

### 3.1. Properties of Novel Ti-xNb-5Ta Alloys

#### 3.1.1. Microstructure Analysis of Ti-xNb-5Ta Alloys

**Figure 1:**
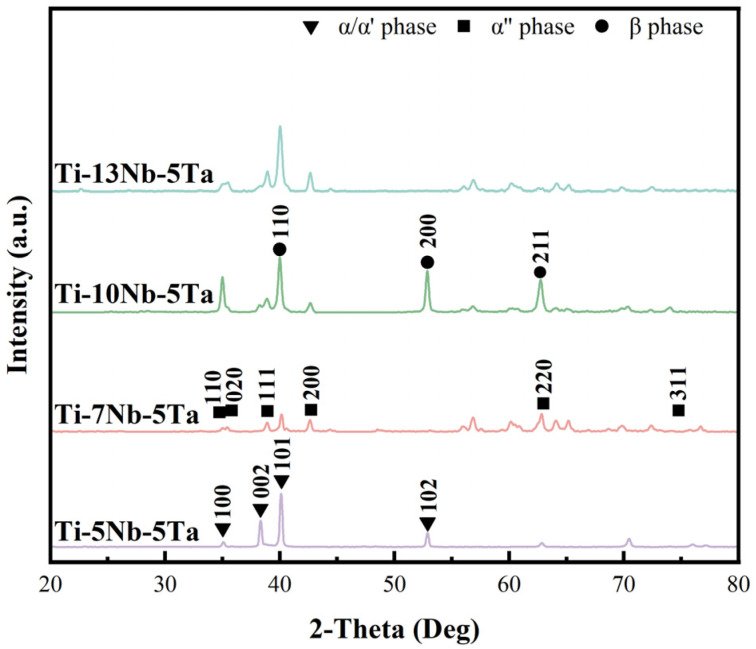
X-ray diffraction pattern.

Figure 1 presents the XRD pattern. The XRD results indicate that the as-cast Ti-xNb-5Ta alloys were primarily composed of α/α’, α”, and β phases, with no other phase structures detected. When the Nb content was 5 wt.%, the alloy predominantly consisted of the α/α’ phase. As the Nb content increased to 7 wt.%, the intensity of the α/α’ phase decreased significantly. In contrast, the α” martensite phase and a small amount of the β phase emerged. At 10 wt.% Nb content, the alloy was mainly characterized by the α” phase, with a reduced α/α’ content and a gradual increase in the β phase. When the Nb content reached 13 wt.%, the α/α’ phase completely transformed into the α” phase and β phase, which is consistent with observations by Talbot et al. [32].

**Figure 2:**
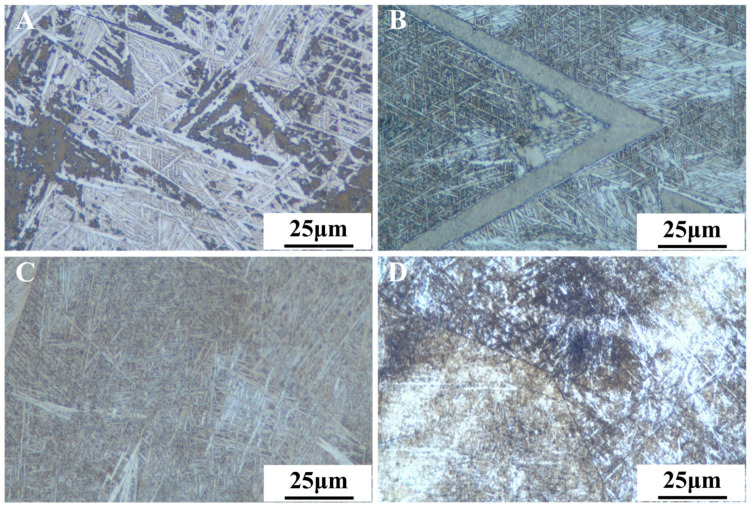
Morphology of (**A**) Ti-5Nb-5Ta, (**B**) Ti-7Nb-5Ta, (**C**) Ti-10Nb-5Ta, and (**D**) Ti-13Nb-5Ta.

Figure 2 shows representative metallographic micrographs of the Ti-xNb-5Ta alloys, revealing a martensitic α″ phase with a short, rod-like morphology in a hypoeutectic composition. As Nb content increased, the grain size decreased from 13 μm for Ti-5Nb-5Ta to 3 μm for Ti-13Nb-5Ta. These metallographic micrographs resemble those observed for Ti-40Nb materials prepared by Manso et al. [33].

#### 3.1.2. Mechanical Properties of Ti-xNb-5Ta Alloy

**Figure 3:**
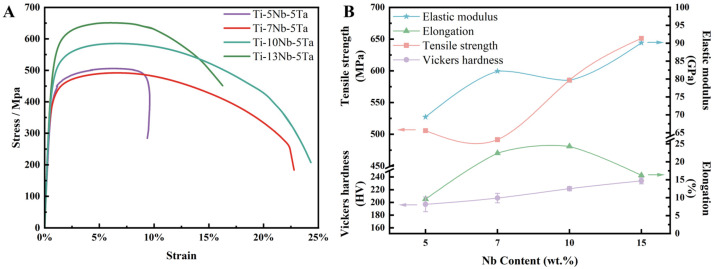
(**A**) Tensile stress–strain curves and (**B**) mechanical properties.

Figure 3A,B display the tensile stress–strain curves and mechanical properties of the Ti-xNb-5Ta alloys, and the accurate mechanical properties are provided in Table 4. Increasing the alloy’s Nb content resulted in a corresponding rise in tensile strength. For Nb contents of 10 wt.% or less, elongation improved with increasing Nb, ranging from 9.59% to 24.29%. However, in the Ti-13Nb-5Ta alloy, which had the highest tensile strength of 651.2 MPa, elongation decreased to 16.23%.

Comparing the Ti-7Nb-5Ta alloy with the Ti-5Nb-5Ta alloy, the decrease in tensile strength was minimal, while elongation significantly increased from 9.59% to 22.40%. With increasing Nb content, the elastic modulus of the alloy increased from 69.4 to 90.1 GPa. However, when the Nb content equaled 10 wt.%, the elastic modulus decreased to 79.6 GPa.

### 3.2. Corrosion Resistance Analysis of Ti-xNb-5Ta Alloy

**Figure 4:**
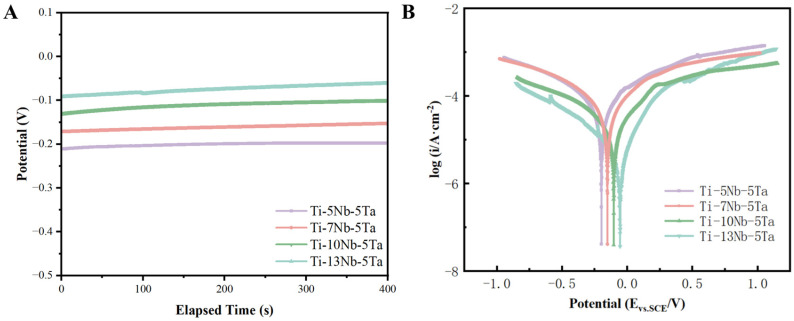
(**A**) Open circuit potential and (**B**) polarization curve.

Electrochemical tests were conducted in SBF to evaluate the corrosion resistance of the Ti-xNb-5Ta alloys. Figure 4A shows the OCP time behavior of the Ti-xNb-5Ta alloys, while Figure 4B presents the representative polarization curves for each sample. The open circuit potentials (E_ocp_), corrosion potentials (E_corr_), and corrosion current densities (i_corr_) derived from Figure 4A,B are summarized in Table 5. The OCP curves for all Ti-xNb-5Ta alloys exhibited a slight increase over 400 s. The polarization curves demonstrated a consistent trend of increasing current density with rising potential in the anodic branches [34,35]. Analysis of the electrochemical parameters revealed that as the Nb content increased, the E_corr_ shifted to more positive values, ranging from −0.196 V to −0.071 V. The i_corr_ values, representing the corrosion rate [36], decreased with higher Nb content, indicating improved corrosion resistance. Notably, the Ti-13Nb-5Ta sample showed the lowest i_corr_ among the four alloys, approximately an order of magnitude smaller than that of the Ti-5Nb-5Ta sample. For comparison, the E_corr_ and i_corr_ values of Cp-Ti were −0.114 V and 115 × 10^−6^A/cm^2^ [37].

**Figure 5:**
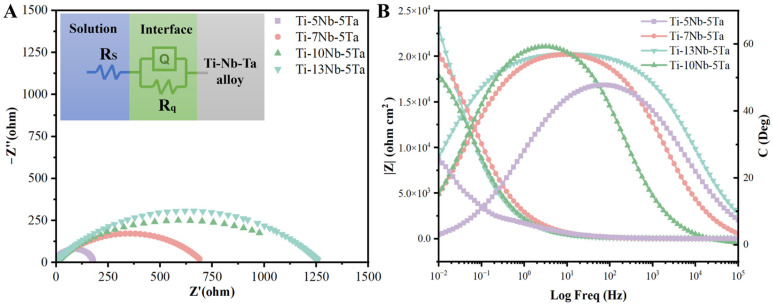
(**A**) Nyquist and (**B**) Bode curves of Ti-xNb-5Ta tested in SBF. The inset is the equivalent circuit used to fit the impedance data.

ElS was employed to analyze the corrosion properties of the Ti-xNb-5Ta alloys, as shown in Figure 5A,B. Nyquist plots for samples immersed in SBF indicated that increasing the Nb content resulted in larger impedance loops, signifying enhanced corrosion resistance at higher Nb concentrations. The inset of Figure 5A illustrates the equivalent circuit used to fit the EIS data. This circuit features a constant phase element, with R_s_ representing the solution resistance, R_q_ denoting the polarization resistance at the interface, and Q serving as the constant phase element [38]. The parameters obtained from fitting the equivalent circuit are summarized in Table 6. The R_s_ values remained consistent across all samples due to the fixed positioning of the working and reference electrodes during measurements. A higher R_q_ value correlates with improved corrosion resistance. Among the tested alloys, the Ti-13Nb-5Ta alloy exhibited the highest polarization resistance, while alloys with a lower Nb content showed reduced corrosion resistance. This trend is further corroborated by the Bode plot presented in Figure 5B.

### 3.3. The Biocompatibility of Ti-xNb-5Ta Alloys

#### 3.3.1. Biomineralization Capacity in an SBF Solution

**Figure 6:**
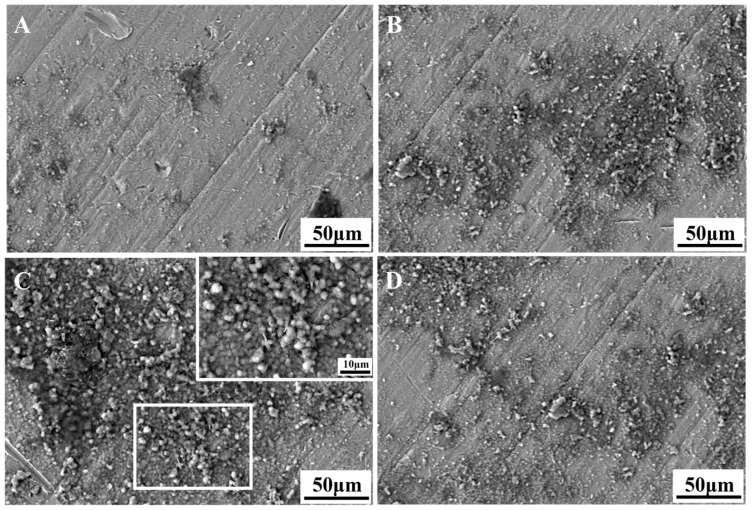
Morphology of the (**A**) Ti-5Nb-5Ta, (**B**) Ti-7Nb-5Ta, and (**C**) Ti-10Nb-5Ta samples, with the inset in the upper right corner showing a higher magnification morphology of the square in (**C**), and (**D**) Ti-13Nb-5Ta samples incubated in SBF for 28 days.

**Figure 7:**
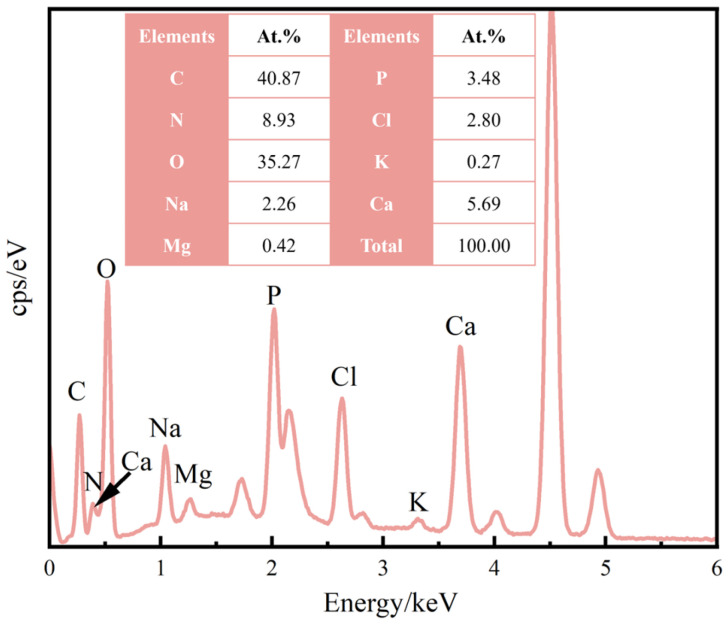
EDS spectra, the inset is the corresponding elemental composition.

In order to assess the surface bioactivity of the biomaterials, the formation of calcium phosphate coating via in vitro SBF immersion is a widely used method [39,40]. Figure 6 presents the surface morphology of the Ti-xNb-5Ta samples after 28 days of immersion in SBF. All samples displayed precipitates on their surfaces following immersion. Notably, the Ti-10Nb-5Ta alloy exhibited substantial surface precipitates (Figure 6C). A magnified view reveals that these precipitates were predominantly spherical, as shown in the inset of Figure 6. Elemental analysis using EDS mapping (Figure 7) confirmed that the precipitate regions were enriched with calcium (Ca) and phosphorus (P), with a measured Ca/P ratio of approximately 1.63, as indicated in the inset of Figure 7. This ratio is close to the Ca/P ratio of human bone tissue (1.67), suggesting the formation of a calcium-deficient apatite precursor on all sample surfaces [40,41].

#### 3.3.2. The Effect of Ti-xNb-5Ta on the Growth and Adhesion of hBMSCs

**Figure 8:**
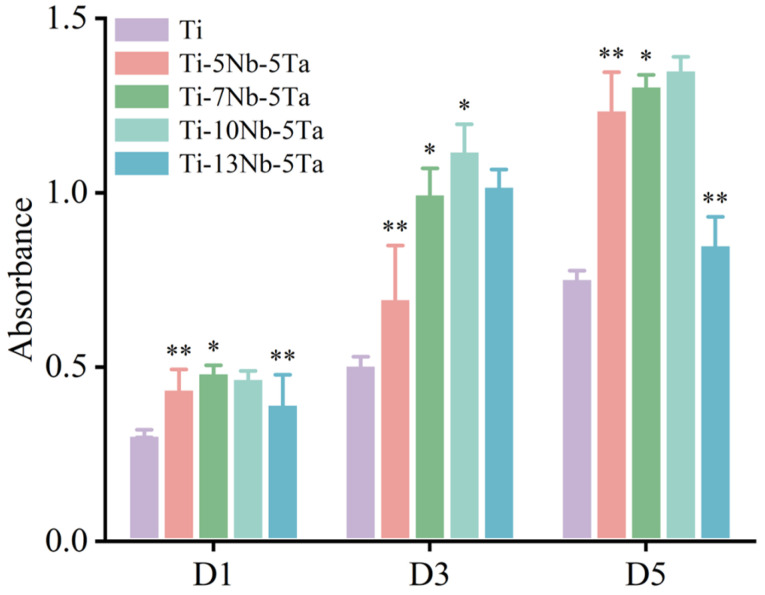
Effects of different Nb contents on hBMSC proliferation in alloys. * represents the inter-group comparison of *p* < 0.05; ** represents the inter-group comparison *p* < 0.001.

**Figure 9:**
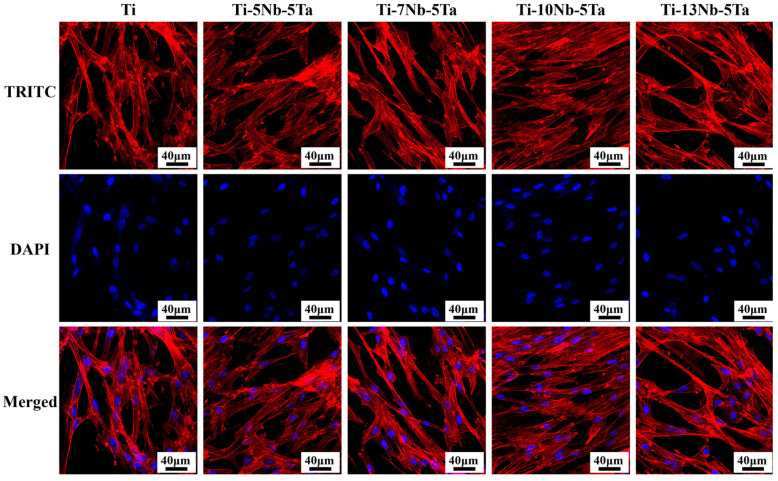
Adhesion images of hBMSCs for alloys with different Nb contents.

The CCK-8 assay results indicated that varying the Nb content enhanced cell proliferation. The cells grew well in all groups during the first three days. However, by day 5, the cell activity in the Ti-13Nb-5Ta group showed a decline (Figure 8). After 5 days of culture, the Ti-10Nb-5Ta alloy demonstrated the highest optical density (OD) value, indicating superior cell proliferation [42]. Immunofluorescent staining was performed to evaluate the morphology of hBMSCs and their nuclei (Figure 9). After 48 h of incubation, DAPI staining labeled the nuclei, while phalloidin was used to stain the cytoskeleton. Confocal microscopy images revealed that cells adhered and grew well on all alloy samples. Notably, cells on the Ti-10Nb-5Ta alloy displayed a larger spreading area and more extended filopodia, whereas cells on the other alloys were predominantly spindle-shaped with smaller spreading areas.

#### 3.3.3. Effect of Ti-xNb-5Ta on ALP Activity of hBMSCs

**Figure 10:**
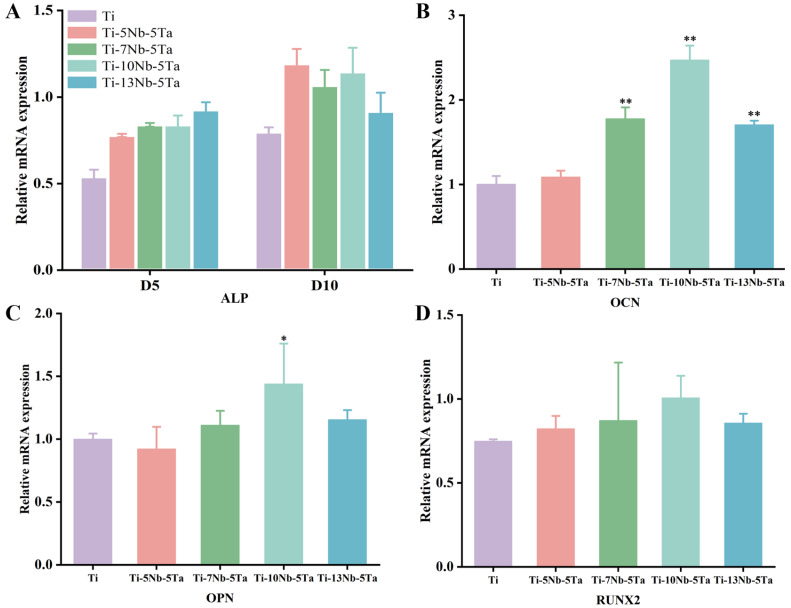
(**A**) Effects of alloys with different Nb contents on the semi-quantification of the ALP expression of hBMSCs. (**B**–**D**) Osteogenic-related mRNA (Ocn, Opn, and Runx2) expression levels. * represents the inter-group comparison of *p* < 0.05; ** represents the inter-group comparison *p* < 0.001.

Semi-quantitative analysis (Figure 10A) showed that on day 5, the Ti-13Nb-5Ta alloy exhibited the highest ALP activity. However, by day 10, the Ti-5Nb-5Ta and Ti-10Nb-5Ta alloys showed significantly elevated ALP activity compared to the other samples. RT-PCR assays were performed to evaluate further osteogenic gene expression in hBMSCs cultured on these alloys. The Ti-10Nb-5Ta alloy displayed higher expression levels of osteogenic genes, including OCN, OPN, and RUNX2, as shown in Figure 10B–D. Gene expression increased with Nb content up to 10 wt.% but declined when the Nb content exceeded this level. These findings suggest that the Ti-xNb-5Ta alloy could enhance the expression of osteogenic genes.

## 4. Discussion

The findings discussed in the previous sections formed the foundation for a detailed evaluation of the Ti-xNb-5Ta alloys investigated in this study. Given the uniformity of arc-melting conditions applied to all Ti-xNb-5Ta alloys, the observed variations in microstructural evolution and mechanical properties could be directly attributed to differences in their chemical composition.

The two compositions with the highest titanium content exhibited notably different phase compositions. This difference was particularly evident in the Ti-7Nb-5Ta alloy, which represented a transition region. The XRD analysis shown in Figure 1 revealed that the dominant phase was the orthorhombic α″ phase, characteristic of martensite. This finding aligns with observations for many other titanium-based alloys, where non-equilibrium martensite dominated the as-built microstructure [43,44]. Its formation is attributed to the rapid cooling induced by the water cooling system during arc-melting.

When the Nb content in the alloy was relatively high, the microstructure predominantly exhibited a fine α″ phase following arc-melting. The resulting solidification structure consisted of small grains, as confirmed by the morphology micrographs in Figure 2. High Nb contents inhibited the epitaxial growth of columnar β phase grains, encouraging microstructure fragmentation due to constitutional supercooling. In the case of the Ti-13Nb-5Ta alloy, the preferred alignment of the plate-like structures can be explained by the strong texture of the parent β phase. Consequently, the columnar grains exhibited a highly uniform orientation. Due to the specific orientation relationships between the parent β phase and the resulting martensite, martensitic features appeared consistent across grains. However, slight variations in the trace angles were noticeable when crossing grain boundaries. Consequently, significant changes in the processing strategy are expected to yield notable variations in microstructural evolution. Ongoing research aims to investigate this phenomenon further and will be addressed in future studies.

Notably, the elongation of Ti-5Nb-5Ta, at less than 15%, fell short of the mechanical property requirements for biomedical metals. When the Nb contents were 10% and 13%, the alloy achieved a tensile strength of 585.3 and 651.2 MPa, respectively. These tensile strengths surpass the TA4G surgical implant standard (580 MPa, GB/T 13810-2017) and that of Ti-17Nb-6Ta (530 MPa) [45], Ti-18Nb (520 MPa) [46], and Ti-3Cu (575 MPa) [47] alloys, while Ti-10Nb-5Ta still maintained an elongation of 24.29%. All self-developed Ti-xNb-5Ta alloys exhibited a lower elastic modulus compared to commonly used titanium alloys such as CP Ti (~104 GPa) [48], TC4 ELI (~117 GPa) [24], and Ti-15Zr (~110 GPa) [49].

As for corrosion resistance, the composition of the alloy’s passive film changed with increasing Nb content [50,51]. When Nb was present in the alloy, passive films such as NbO, NbO_2_, and Nb_2_O_5_ formed [52,53]. As the Nb content increased, the proportion of Nb_2_O_5_ passive film rose while the proportion of Ti_2_O_3_ decreased, thereby enhancing the corrosion resistance of the alloy. The Nb incorporated into the alloys could stabilize the passive film, improving the alloy’s corrosion resistance [54]. The next step can further explore the antibacterial ability of the alloy’s passive film.

The findings from the biomineralization capacity, CCK-8 assay, ALP semi-quantitative analysis, and RT-PCR indicated that the Ti-xNb-5Ta alloy enhanced the early osteogenic differentiation of hBMSCs better than Ti, indicating that the addition of Nb can effectively enhance the biocompatibility of Ti alloys, which is consistent with the findings of Du et al. [55]. Future studies could explore the relationship between Nb content and osteogenic performance.

The material under study was developed specifically for the biomedical field, especially for dental implants. We believe this series of materials can reduce failure rates from both the implant and bone side due to their strong mechanical properties and better biocompatibility. In the following step, we will conduct in vivo experiments on beagle dogs in order to unveil the novel material’s failure rate. Further research will explore the improvement of materials using new processing technology. For example, additive manufacturing can further match stiffness with human bone. Chmielewska et al. summarized a series of porous scaffolds fabricated by additive manufacturing, which provides a direction for the follow-up research of this material [56].

## 5. Conclusions

In this study, Ti-xNb-5Ta near-β-type titanium alloys were successfully fabricated and exhibited superior mechanical properties and biocompatibility suitable for surgical implants. These alloys meet the TA4G surgical implant standards, offering improved elongation and reduced elastic modulus. The key findings include the following:

1. Microstructure: The Ti-xNb-5Ta alloys predominantly displayed an orthorhombic α″ phase with a martensitic microstructure. Increasing the Nb content reduced the grain size from 13 μm (Ti-5Nb-5Ta) to 3 μm (Ti-13Nb-5Ta).
2. Mechanical Properties: The Ti-xNb-5Ta alloys’ mechanical properties (higher tensile strength and lower elastic modulus) demonstrated potential as biomedical materials, requiring further investigations such as heat treatment or additive manufacturing.
3. Corrosion Resistance: The EIS of the Ti-xNb-5Ta alloy demonstrated that as the Nb content increased, the corrosion resistance increased. All the Ti-xNb-5Ta alloys exhibited superior corrosion resistance compared to existing biomedical materials.
4. Biocompatibility: Ti-xNb-5Ta alloys have the possibility to enhance the early osteogenic differentiation of hBMSCs. In future studies, we will conduct in vivo experiments on, for example, osseointegration, vascularization, and possible inflammatory responses to further explore the potential of Ti-xNb-5Ta alloys as biomedical materials.

Conclusively, this study provides a series of new materials for narrow-diameter dental implants that have outstanding properties compared with existing medical materials. However, these properties can be improved further through heat treatment or additive manufacturing to challenging more extreme conditions and, as such, may require a compromise between both tensile strength and elongation properties when optimizing a given set of alloying elements.

## Figures and Tables

**Table 1:** Actual composition of the alloys (wt.%).

Alloys	Nb	Ta	C	N	H	O	Ti
Ti-5Nb-5Ta	5.18	5.21	0.014	0.007	0.002	0.013	Bal.
Ti-7Nb-5Ta	7.13	4.96	0.016	0.006	0.002	0.015	Bal.
Ti-10Nb-5Ta	9.95	5.18	0.017	0.027	0.001	0.016	Bal.
Ti-13Nb-5Ta	13.16	5.08	0.023	0.018	0.002	0.019	Bal.

**Table 2:** SBF composition (all chemicals from Sinopharm, Shanghai, China).

Composition	Concentration, g/L
NaCl	8.035
NaHCO_3_	0.355
KCl	0.255
K_2_HPO_4_·3H_2_O	0.231
MgCl_2_·6H_2_O	0.311
CaCl_2_	0.292
Na_2_SO_4_	0.072
Tris	6.118

**Table 3:** Nucleotide sequences of primers used for RT-PCR.

Gene	Forward Primer (5′-3′)	Reverse Primer (5′-3′)
Ocn	GGCGCTACCTGTATCAATGG	GTGGTCAGCCAACTCGTCA
Opn	TCCTAGCCCCACAGACCCTT	CACACTATCACCTCGGCCA
Runx2	GCCGGGAATGATGAGAACTA	GCCGGGAATGATGAGAACTA

**Table 4:** Ti-xNb-5Ta mechanical properties.

Nb Content	Elastic Modulus/GPa	Tensile Strength/MPa	Elongation/%	Vickers Hardness/HV
5 Nb	69.4	505.7	9.59	197.0 ± 11.6
7 Nb	82.2	491.5	22.40	206.8 ± 7.4
10 Nb	79.6	585.3	24.29	221.6 ± 3.3
13 Nb	90.1	651.2	16.23	233.9 ± 4.8

**Table 5:** Electrochemical data of Ti-xNb-5Ta alloys obtained from OCP and polarization curves.

Nb Content	E_ocp_ (V)	E_corr_ (V)	i_corr_ (10^−6^ A/cm^2^)
5 Nb	−0.19	−0.196	8.71
7 Nb	−0.16	−0.151	7.59
10 Nb	−0.11	−0.103	3.62
13 Nb	−0.07	−0.071	0.97

**Table 6:** Electrochemical impedance parameters obtained by model R(QR) of the equivalent circuits for Ti-xNb-5Ta alloys.

Nb Content	R_S_ (Ω·cm^2^)	Q (F·cm^−2^)	R_q_ (Ω·cm^2^)	n_q_
5 Nb	0.43	6.19 × 10^−4^	189	0.8
7 Nb	1.06	2.44 × 10^−4^	788	0.595
10 Nb	1.69	2.53 × 10^−4^	1009	0.7198
13 Nb	1.01	3.56 × 10^−4^	1225	0.6119

## Data Availability

The original contributions presented in this study are included in the article. Further inquiries can be directed to the corresponding author.
